# Preclinical modeling of combined phosphatidylinositol-3-kinase inhibition with endocrine therapy for estrogen receptor-positive breast cancer

**DOI:** 10.1186/bcr2833

**Published:** 2011-03-01

**Authors:** Cesar G Sanchez, Cynthia X Ma, Robert J Crowder, Therese Guintoli, Chanpheng Phommaly, Feng Gao, Li Lin, Matthew J Ellis

**Affiliations:** 1 Department of Hematology-Oncology, School of Medicine, Pontificia Universidad Catolica de Chile, Lira 85, 4th floor, Santiago 8330023, Chile; 2 Division of Oncology, Department of Medicine, Washington University in St Louis, 660 S Euclid Avenue, Campus Box 8069, St Louis, MO 63110, USA; 3 Siteman Comprehensive Cancer Center, 660 S Euclid Avenue, Campus Box 8100, St Louis, MO 63110, USA; 4 Division of Biostatistics, Department of Medicine, Washington University in St Louis, 660 S Euclid Avenue, Campus Box 8067, St Louis, MO 63110, USA

## Abstract

**Introduction:**

Inhibition of phosphatidylinositol-3-kinase (PI3K) induces apoptosis when combined with estrogen deprivation in estrogen receptor (ER)-positive breast cancer. The aims of the present study were to identify effective PI3K pathway inhibitor and endocrine therapy combinations, to evaluate the effect of PI3K pathway mutations and estrogen dependency on tumor response, and to determine the relevance of *PIK3CA *mutation in recurrent disease.

**Methods:**

The PI3K catalytic subunit inhibitor BKM120, the mammalian target of rapamycin (mTOR) inhibitor RAD001 and the dual PI3K/mTOR inhibitor BGT226 were tested against ER-positive breast cancer cell lines before and after long-term estrogen deprivation (LTED). The impact of estradiol deprivation and the ER downregulator fulvestrant on PI3K pathway inhibitor-induced apoptosis was assessed. *PIK3CA *hotspot mutation analysis was performed in 51 recurrent or metastatic breast cancers and correlated with ER status and survival.

**Results:**

Drug-induced apoptosis was most marked in short-term estrogen-deprived cells with *PIK3CA *mutation and phosphatase and tensin homolog loss. Apoptosis was most highly induced by BGT226, followed by BKM120, and then RAD001. Estradiol antagonized PI3K inhibitor-induced apoptosis following short-term estrogen deprivation, emphasizing a role for estrogen-deprivation therapy in promoting PI3K inhibitor activity in the first-line setting. ER-positive MCF7 LTED cells exhibited relative resistance to PI3K pathway inhibition that was reversed by fulvestrant. In contrast, T47D LTED cells exhibited ER loss and ER-independent PI3K agent sensitivity. *PIK3CA *mutation was prevalent in relapsed ER-positive disease (48%) and was associated with persistent ER positivity and a late relapse pattern.

**Conclusions:**

Estrogen deprivation increased the apoptotic effects of PI3K and dual PI3K/mTOR inhibitors in ER-positive disease, providing a rationale for PI3K/aromatase inhibitor combinations as first-line therapy. In LTED cells, differential effects on ER expression may be a relevant consideration. When ER was persistently expressed, fulvestrant strongly promoted PI3K drug activity. When ER was lost, PI3K inhibitor monotherapy was sufficient to induce high-level apoptosis. Although tumors with *PIK3CA *mutation had a late recurrence pattern, these mutations were common in metastatic disease and were most often associated with persistent ER expression. Targeting *PIK3CA *mutant tumors with a PI3K pathway inhibitor and fulvestrant is therefore a feasible strategy for aromatase-inhibitor-resistant ER-positive relapsed breast cancer.

## Introduction

Since the widespread adoption of tamoxifen, modest improvements in patient outcomes have been observed in estrogen receptor (ER)-positive breast cancer patients through the introduction of aromatase inhibitors and fulvestrant, but prognosis remains poor for many patients [1] due to *de novo *or acquired endocrine therapy resistance. A major biological barrier to successful treatment of ER-positive disease is that endocrine treatment induces cell cycle arrest but not high-level cell death [2,3]. Disseminated ER-positive breast cancer cells therefore persist, acquire endocrine therapy resistance and cause disease progression and death. An ideal regimen for ER-positive disease would effectively delete ER-positive cells, thereby circumventing secondary resistance and obviating the requirement for long-term endocrine treatment with its attendant quality-of-life detriment, chronic toxicity and expense.

Targeting the pro-survival phosphatidylinositol-3-kinase (PI3K) signaling is intriguing in this regard. Genes in the PI3K pathway are frequently mutated or amplified in ER-positive breast cancer, suggesting that hyperactivation of PI3K signaling is a key target that, if effectively inhibited, could improve outcomes [4]. We have already shown that estrogen deprivation in combination with PI3K inhibition by RNA interference induces synthetic lethality and promotes cell death in ER-positive breast cancer cell lines [5], providing a rational for combination approaches that target the ER and PI3K pathways simultaneously. ER-positive breast cancers are genetically heterogeneous, however, and cell-intrinsic factors may modulate sensitivity to this approach. It is unclear whether mutations in PI3K pathway proteins - especially in *PIK3CA*, the gene that encodes the PI3Kα catalytic subunit - sensitize tumors to this strategy. Furthermore, the optimal combinations of endocrine agents and PI3K pathway inhibitors have not been established and the strategy for patients with estrogen deprivation (aromatase inhibitor)-resistant disease is unclear. Finally, a question has recently arisen regarding the relevance of the common *PIK3CA *mutation as a therapeutic target since several reports have suggested that *PIK3CA *mutation is associated with a favorable prognosis [6,7]. If this is the case, *PIK3CA *mutations would be expected to be rare in advanced disease and therefore less relevant as a therapeutic target in this setting.

To address these issues, a panel of ER-positive breast cancer cell lines with different PI3K pathway mutations were tested against three different PI3K pathway inhibitors, with selectivity against either the rapamycin-sensitive mammalian target of rapamycin (mTOR) complex (Everolimus/RAD001), the PI3K catalytic isoforms (BKM120) or both PI3K and mTOR (BGT226) in the presence or absence of estrogen or ER downregulation by fulvestrant. In addition, these inhibitor combinations were retested after the development of long-term estrogen deprivation (LTED) resistance to model-acquired resistance to estrogen deprivation. *PIK3CA *mutation analysis was performed on tumor biopsies from recurrent disease and in patients with stage 4 breast cancer to determine the prevalence of mutations in advanced disease and to correlate mutation status with the rate of tumor progression and death.

## Materials and methods

### Pharmacological agents

BGT226, BKM120 and RAD001 were obtained through material transfer agreements with Novartis (Basle, Switzerland). Fulvestrant (Sigma-Aldrich, St. Louis, MO, USA), LY294002 (Enzo Life Sciences, Plymouth Meeting, PA, USA), rapamycin (Enzo Life Sciences) and 17β-estradiol (Sigma-Aldrich) were from commercial sources. 17β-Estradiol was dissolved in ethanol; inhibitors were dissolved in dimethylsulfoxide.

### Cell culture

The HCC712 cell line [8] was kindly provided by Dr Adi Gazdar. Other cell lines were obtained from American Type Culture Collection (Manassas, VA, USA). Experiments with parental cell lines were performed with low-passage-number cells used within 2 to 3 months following revival from the supplier. Cell lines were propagated in RPM1 1640 containing 10% fetal bovine serum (FBS) with antibiotic and supplements (50 μg/ml gentamycin, pyruvate, 10 mM Hepes and glucose to 4.5 g/l) in a humidified 37°C incubator containing 5% carbon dioxide. LTED MCF7 and T47D cell line variants were produced by culturing the parental lines for >9 months in phenol-red-free RPMI 1640 containing 5% charcoal-stripped FBS (charcoal-stripped serum (CSS); Invitrogen, Carlsbad, CA, USA) containing antibiotic and supplements (CSS medium). Estrogen-retreated LTED sublines (LTED-R cells) were created by treating LTED cells growing in CSS medium with 10 nmol/l 17β-estradiol for at least 4 months prior to experiments. For studies using short-term estrogen deprivation (STED) parental cell lines, cells were maintained in CSS medium for 1 to 3 weeks prior to experimental treatments.

### Protein extraction

For pharmacological treatments, cells were deprived of serum for 3 to 4 hours, pretreated with the indicated agents for 20 minutes, and then treated with or without 20% FBS for 15 minutes. Lysates were prepared by extracting cells in lysis buffer as previously described [5].

### Immunoblotting

Extracted proteins were analyzed by immunoblotting as previously described [5] using primary antibodies and appropriate horseradish peroxidase-conjugated secondary antibodies (1:20,000; Jackson Immmunoresearch Laboratories, West Grove, PA). Primary antibodies for immunodetection included: ER (RM-9101; Fisher Scientific, Fremont, CA, USA), human epidermal growth factor receptor 2 (HER2) (#A0485; Dako, Carpenteria, CA, USA), phospho-Y1248-HER2 (#M7269; Dako), p110δ (ab1678; AbCam, Cambridge, MA, USA) and actin (sc-1616; Santa Cruz Biotechnology, Santa Cruz, CA, USA). Antibodies for detecting p110α (#4249), p110β (#3011), p110γ (#4252), phosphatase and tensin homolog (PTEN) (#9559), Akt1 (#2938), Akt2 (#2964), Akt3 (#3788), phospho-Ser473 Akt (p-Akt) (#4060), mTOR (#2983), S6 protein kinase 1 (#2708), phospho-Thr 389-S6 protein kinase 1 (#9206), S6 (#2217), phospho-Ser235/236 S6 (p-S6) (#4856), p44/42 mitogen-activated protein kinase (MAPK; ERK1/2) (#4695) and phospho-Thr202/Tyr204 p44/42 MAPK (p-ERK1/2) were from Cell Signaling Technology (Danvers, MA, USA).

### Cell growth assay and calculation of 50% inhibitory/lethal concentrations

To determine the effects of estradiol and fulvestrant on the growth of LTED cells, the cells growing in CSS medium were plated in 96-well Optilux dishes and were treated without or with fulvestrant (300 nmol/l) or the indicated concentrations of 17β-estradiol on the day after plating. The medium was replenished every 3 to 4 days and cell growth was assessed after 7 days by measuring Alamar Blue reduction (555λ_Ex_/585λ_Em_) with a fluorescent microplate reader. For calculation of the half maximal inhibitory concentration (IC_50_) and the 50% lethal concentration (LC_50_), cells were cultured in phenol-red-free RPM1 1640 containing 5% CSS (CSS medium) for at least 1 week prior to plating in 96-well Optilux dishes (~1,000 to 3,000 cells/well per given cell line) for drug treatment. Alternatively, cells growing in phenol red RPMI 1640 medium containing 10% FBS were plated in 96-well Optilux dishes and then switched to CSS medium for at least 1 week prior to drug treatment. Five dilutions of each drug were made using a 1:5 serial dilution. Treatments were performed in triplicate and the experiments in each cell line were performed at least twice. The effect of treatments on cell viability were assessed 0 hours (at the time of drug addition) and 96 hours after drug exposure by measuring the Alamar Blue reduction (555λ_Ex_/585λ_Em_) using a fluorescent microplate reader. Cell growth was analyzed using GraphPad Prism version 5.00 for Windows (GraphPad Software, San Diego, CA, USA). The fitted curves were then used to determine the IC_50 _and LC_50 _values.

### Apoptosis assay

To quantify apoptosis, cells growing in CSS medium were treated as indicated for 4 days. For treatments using fulvestrant, cells were pretreated with fulvestrant for 3 days prior to treatment with estradiol or PI3K inhibitors to ensure sufficient downregulation of the ER. Floating and adherent cells were then collected and labeled to detect apoptotic cells using the APO-BrdU TUNEL Assay Kit (Invitrogen) in accordance with the manufacturer's instructions. For each sample, a minimum of 10,000 events were acquired on a Cytomics FC500 flow cytometer (Becton Dickinson, Fremont, CA, USA) and data were analyzed using FlowJo software (Tree Star, Ashland, OR, USA).

### Patient samples

Human tumor samples from patients with recurrent or metastatic breast cancer were obtained under the auspices of an Institutional Review Board-approved protocol at the Siteman Cancer Center at Barnes-Jewish Hospital and Washington University School of Medicine between January 2004 and January 2009. Informed consent was obtained from all patients involved. Information on ER, progesterone receptor and HER2 at initial and recurrent diagnosis was obtained from patient pathological reports. Preparation of samples for tumor DNA extraction and resequencing of *PIK3CA *exons 9 and 20 using genomic DNA was performed as described previously [5].

### Statistical analysis

Unless indicated otherwise, quantitative data for *in vitro *studies are presented as the mean ± standard deviation. The effect of pharmacologic treatments on apoptosis was analyzed using analysis of variance, and *post-hoc *multiple comparisons were performed between specific treatments if the overall difference reached statistical significance (*P *< 0.05). The relationship between *PIK3CA *mutation and other covariates was performed using Fisher's exact test or Student's *t *test as appropriate. Overall survival was defined as the time from diagnosis to the date of death due to any cause. Survivors were censored at the date of last contact. Disease-free survival was only calculated in subjects with an initial stage of I to III and was defined as the time from diagnosis to the first recurrence or death. The overall survival and disease-free survival across mutation status were estimated using the Kaplan-Meier product limit method and were compared by log-rank test. All analyses were two-sided and significance was set at *P *< 0.05. Statistical analyses were performed using SAS software (SAS Institute, Cary, NC, USA).

## Results

### Expression and activation of PI3K pathway proteins in breast cancer cells

**Figure 1:**
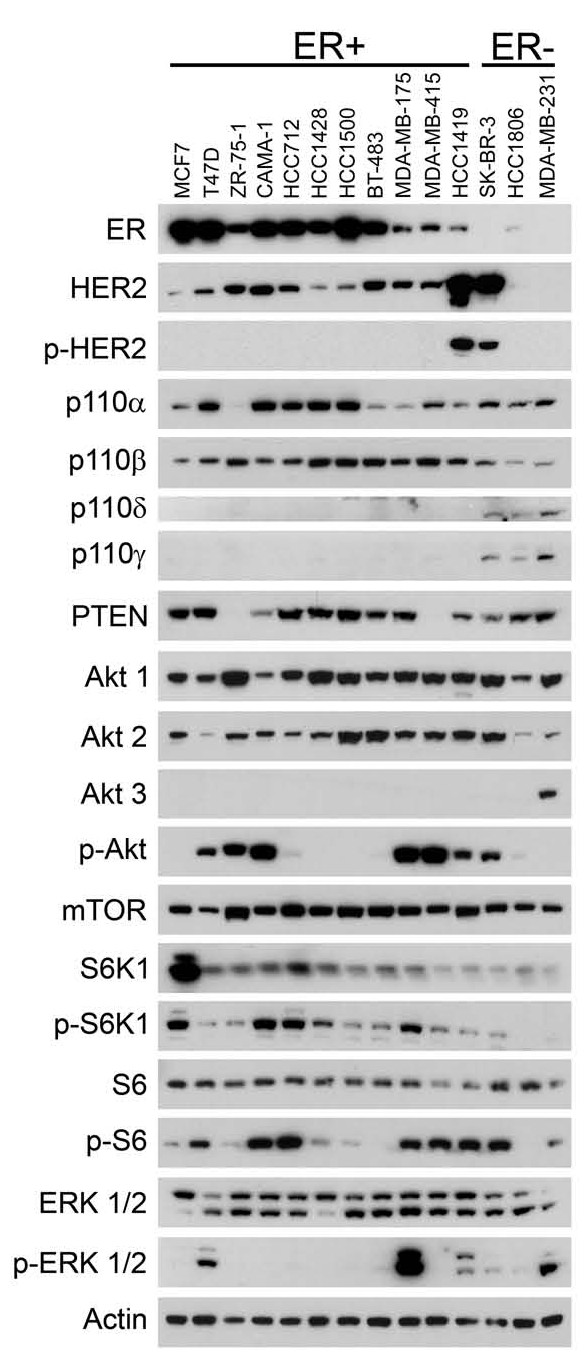
**Analysis of phosphatidylinositol-3-kinase pathway signaling in breast cancer cells**. Cell lines were grown to subconfluency and placed in medium containing low (0.5%) fetal bovine serum overnight prior to preparation of cell lysates. Equal amounts (25 μg) of extracted protein from each cell line were then immunoblotted using antibodies against the indicated proteins. P-Tyr1248 HER2 (*p-HER2*); p-Ser473 Akt (*p-Akt*); p-Thr389 S6 protein kinase 1 (*p-S6K1*); p-Ser235/236 S6 (*p-S6*); p-Thr202/Tyr204 ERK (*p-ERK*). ER, estrogen receptor; HER2, human epidermal growth factor receptor 2; PTEN, phosphatase and tensin homolog; mTOR, mammalian target of rapamycin.

To assess PI3K signaling activity in the panel of breast cancer cells used for the present investigation, the levels of phosphorylated forms of AKT, S6 protein kinase 1 and S6 (indicators of PI3K signaling activation), and the expression of PI3K catalytic subunit isoforms, PTEN, AKT isoforms and mTOR were examined (Figure 1). The panel included ER-positive breast cancer cells with activating *PIK3CA *mutations (helical domain mutation: MCF7 and BT-483; kinase domain mutation: T47D), *PTEN *mutation (MDA-MB-415, ZR75-1 and CAMA-1), *HER2 *gene amplification (HCC1419) or wild-type *PIK3CA *and *PTEN *(HCC712, HCC1428, HCC1500 and MDA-MB-175), and ER-negative breast cancer cell lines with *HER2 *amplification (SK-BR-3), and wild-type *PIK3CA *and *PTEN *(HCC1806). The ER-negative MDA-MB-231 cell line is wild-type for *PIK3CA *and *PTEN *but harbors mutations in *K-RAS *and *B-RAF*.

While the PI3K p110α and p110β catalytic subunits were present in all cell lines, the PI3K p110δ and p110γ catalytic subunits were significantly expressed only in ER-negative (SK-BR-3, HCC1806 and MDA-MB-231) cell lines. Akt1 and Akt2 were expressed in all tested breast cancer cell lines, but Akt3 was detectable only in MDA-MB-231 cells [9]. Consistent with previous studies, high levels of p-Akt were present in cells with *PIK3CA *kinase domain mutation (T47D), *PTEN *mutation (MDA-MB-415, ZR75-1 and CAMA-1), *HER2 *amplification (HCC1419, SK-BR-3) [9-11] and the heregulin-dependent MDA-MB-175 cell line. Phosphorylation of the PI3K downstream target S6 closely paralleled Akt phosphorylation.

These data indicate that mutations in *PIK3CA *and *PTEN *or amplification of *HER2 *are associated with PI3K pathway activation in breast cancer.

### BGT226, BKM120 and RAD001 inhibit PI3K pathway signaling in breast cancer cells

There are at least four general subcategories of PI3K pathway inhibitors, based upon target specificity, that are currently in clinical use or in various phases of clinical testing. These include inhibitors of PI3K catalytic subunits; inhibitors of the Akt serine-threonine kinase; inhibitors of mTOR; and multi-targeted agents, which typically have dual-specificity PI3K and mTOR kinase inhibitors [12]. This paper focuses on three of these four classes of agent: RAD001 (inhibitor against rapamycin-sensitive mTOR complex), BKM120 (inhibitor against PI3K catalytic isoforms) and BGT226 (dual inhibitor of PI3K/mTOR).

**Figure 2:**
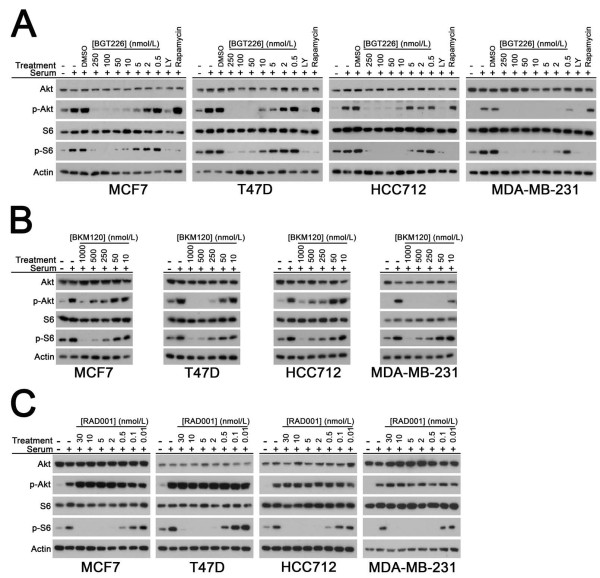
**BGT226, BKM120 and RAD001 inhibit phosphatidylinositol-3-kinase pathway signaling in breast cancer cells**. Western blots showing effects of **(a) **BGT226, **(b) **BKM120 and **(c) **RAD001 dose escalation on p-Ser473 Akt (*p-Akt*) and p-Ser235/236 S6 (*p-S6*) in breast cancer cell lines. Cells were stimulated with 20% fetal bovine serum (15 minutes) in the presence of solvent (dimethylsulfoxide (DMSO), BGT226-treatment only) or the indicated concentrations of phosphatidylinositol-3-kinase (PI3K) inhibitors. LY294002 (LY, 20 μmol/l) and rapamycin (100 nmol/l) were used as controls in the BGT226 treatment panel for total PI3K inhibition (LY) and mammalian target of rapamycin inhibition (rapamycin). Total Akt, S6 and actin are shown as loading controls. Representative results obtained in at least two experiments per cell line per drug treatment are shown.

To illustrate the inhibitory activities of BGT226, BKM120 and RAD001 on PI3K pathway signaling, the phosphorylation levels of Akt (p-Akt) and S6 (p-S6) were assessed by western blotting in MDA-MB-231, MCF7, T47D, or HCC712 cell lines in the presence of increasing dose of drug. As expected, BGT226 and BKM120 inhibited the phosphorylation of both Akt and S6 in all tested lines (Figure 2a,b). BGT226 treatment produced almost complete inhibition of PI3K signaling at low nanomolar (50 nmol/l) concentrations, indicating a similar, or greater, potency compared with that of the dual PI3K/mTOR inhibitor BEZ235 [5,13,14]. In contrast, significant inhibition of PI3K signaling following BKM120 treatment occurred in the mid-nanomolar to high-nanomolar concentration range (250 to 1,000 nmol/l) in most cell lines. In all cell lines, RAD001 treatment completely inhibited S6 phosphorylation at low nanomolar (5 nmol/l) concentrations, with the paradoxical increase in Akt phosphorylation MCF7 cells already noted by other investigators (Figure 2c) [14-16].

These data indicate that PI3K pathway inhibitors effectively suppressed their respective targets regardless of individual differences in PI3K pathway mutation status.

### *PIK3CA *mutation sensitizes short-term estrogen-deprived ER-positive breast cancer cells to PI3K pathway inhibitors

**Figure 3:**
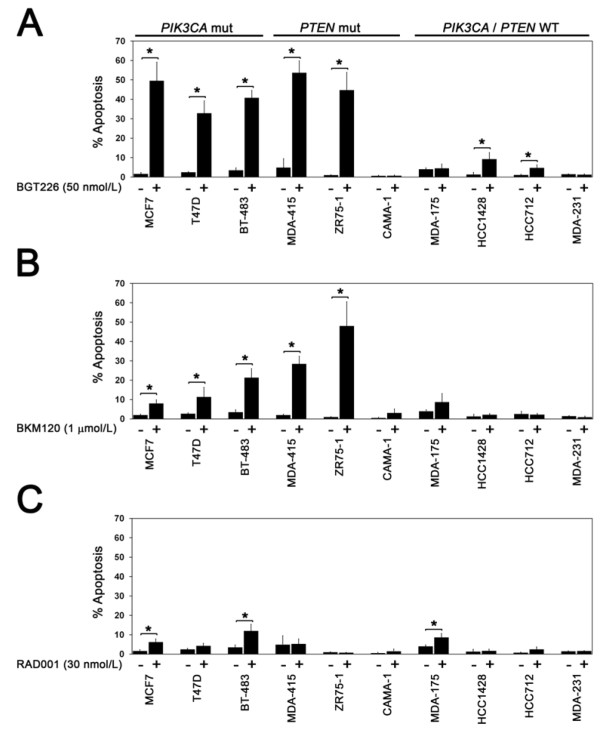
***PIK3CA *mutation and phosphatase and tensin homolog loss confer sensitivity to phosphatidylinositol-3-kinase pathway inhibitors**. *PIK3CA *mutation and phosphatase and tensin homolog (PTEN) loss confer sensitivity to phosphatidylinositol-3-kinase pathway inhibitors in estrogen-deprived estrogen-receptor-positive breast cancer cells. Cells cultured under estrogen-deprived conditions were treated with **(a) **50 nmol/l BGT226, **(b) **1 μmol/l BKM120 or **(c) **30 nmol/l RAD001 and apoptosis was measured after 4 days of treatment by TUNEL flow cytometry. Results are from at least three replicates for each treatment condition per cell line. Significant induction of apoptosis by treatment with inhibitors is indicated (*P < 0.05). WT, wild-type.

To extend our previous observations regarding the sensitizing effect of estrogen deprivation on the apoptotic effect of PI3K pathway inhibitors in ER-positive breast cancer [5], a larger panel of ER-positive breast cancer cell lines was examined that varied with respect to *PIK3CA *and *PTEN *mutation status (Figure 3). Cells in the panel were acutely deprived of estrogen for 1 to 3 weeks prior to treatment with BGT226, BKM120 or RAD001 at concentrations that were found to be sufficient to abrogate pathway signaling (Figure 2a to 2c). The MDA-MB-231 line served as a control for off-target inhibitor effects since this line does not undergo apoptosis when treated with the dual PI3K/mTOR inhibitor BEZ235 [5,17] or combined siRNA knockdown of *PIK3CA *and *PIK3CB *[5].

Induction of apoptosis was measured by TUNEL assay after treatment with BGT226 (50 nmol/l), BKM120 (1 μmol/l) or RAD001 (30 nmol/l) (Figure 3a to 2c). In the absence of estrogen, BGT226 treatment induced the highest levels of apoptosis, followed by BKM120, whereas RAD001 treatment produced only a modest increase in apoptosis in a few cell lines (Figure 3a to 2c), suggesting this class of agent may be a relatively ineffective partner for endocrine therapy combinations. Importantly, we observed that the induction of high levels of apoptosis by both BGT226 and BKM120 was restricted to *PIK3CA *mutant lines (MCF7, T47D and BT-483) and the PTEN-negative MDA-MB-415 and ZR75-1 cell lines. BGT226 treatment also produced a significant but modest increase in apoptosis in the HCC1428 line (wild-type *PIK3CA *and *PTEN*) and the *PIK3CB*-amplified HCC712 cell line, compatible with this agent having the broadest inhibitory activity. Sensitivity to PI3K pathway inhibition and the presence of a pathway mutation, however, were not linked in all lines because *PTEN *mutant CAMA-1 cells were resistant to BGT226 and BKM120 (Figure 3a,b) despite effective inhibition of PI3K pathway signaling (data not shown). Interestingly, the absence of ERK1/2 phosphorylation (Figure 1) in CAMA-1 argues against the activation of the ERK pathway as a mechanism of resistance. The effect of RAD001 on apoptosis was modest overall, but two of the three cell lines in which RAD001 induced apoptosis (MCF7, BT-483) contain *PIK3CA *helical domain mutations.

Taken together, these data indicate that dual PI3K/mTOR and PI3K isoform inhibitors are likely to produce the greatest effects in ER-positive breast cancer, particularly in tumors harboring *PIK3CA *mutation and, possibly, PTEN loss.

As a complementary approach for measuring relative drug sensitivity, the IC_50 _and LC_50 _values were calculated for all three inhibitors in the cell line panel under estrogen-deprived conditions (Table 1). Consistent with TUNEL assay results, LC_50 _values in the low nanomolar per liter range were obtained in the PTEN-negative MDA-MB-415 and ZR75-1 lines and in the three *PIK3CA *mutant (MCF7, T47D, BT-483) cell lines. The LC_50 _values for BKM120 were higher than for BGT226, which is consistent with the higher concentration of BKM120 needed to inhibit PI3K signaling in cell lines (Figure 2b). As expected, BKM120-sensitive cell lines identified by TUNEL generally exhibited lower LC_50 _values. Although the LC_50 _value for RAD001 was attained in HCC1428 cells, we did not observe any induction of apoptosis by TUNEL assay (Figure 3c). Regardless, the data for IC_50 _and LC_50 _were mostly consistent with results obtained from TUNEL assays.

**Table 1:** Determination of LC_50 _and IC_50 _values for BGT226, BKM120 and RAD001 in breast cancer cells

Cell line	ER status	Genotype	BGT226 (nmol/l)		BKM120 (nmol/l)		RAD001 (nmol/l)	
			LC_50_	IC_50_	LC_50_	IC_50_	LC_50_	IC_50_
MCF7	Positive	PIK3CA E545K	7.5	3.5	3,981	248	>625	>625
T47D	Positive	PIK3CA H1047R	10	2.7	316	128	>625	1.5
HCC712	Positive	PIK3CB amp	549	>625	>10,000	347	>625	>625
MCF7 LTED	Positive	PIK3CA E545K	398	1.18	2,691	70.7	>625	<1
MCF7 LTED-R	Positive	PIK3CA E545K	617	5.1	>10,000	4,926	>625	>625
T47D LTED	Negative^a^	PIK3CA H1047R	19	2.3	630	243	>625	<1
BT-483	Positive	PIK3CA E542K	2.5	7.05	>10,000	>10,000	>625	<1
MDA-MB-415	Positive	PTEN mut	28.1	<1	1,584	1,294	>625	>625
CAMA-1	Positive	PTEN mut	275	46.2	>10,000	>10,000	>625	<1
ZR75-1	Positive	PTEN mut	1.3	<1	363	207	>625	<1
HCC1428	Positive	PIK3CA/PTEN wt	501	>625	1,258	1,138	3.1	<1
MDA-MB-175	Positive	PIK3CA/PTEN wt	>625	<1	5,011	>10,000	>625	<1
MDA-MB-231	Negative	K-Ras, B-Raf mut	>625	<1	>10,000	1,237	>625	>625

Cell lines growing under estrogen-deprived conditions in CSS medium were incubated with solvent control or increasing concentrations of the indicated compounds and cell viability was assessed at 0 hours (time of drug addition) and at 96 hours after treatment. *PIK3CA*, *PIK3CB *and *PTEN *mutation information has been published previously [5,22] or was obtained from the Sanger website [31]. ER, estrogen receptor; IC_50_, half maximal inhibitory concentration; LC_50_, 50% lethal concentration; mut, mutant; wt, wild-type. ^a^ER was not detectable by western blot.

### Estradiol inhibits BGT226 and BKM120 treatment-induced apoptosis but in a cell-line-dependent manner

**Figure 4:**
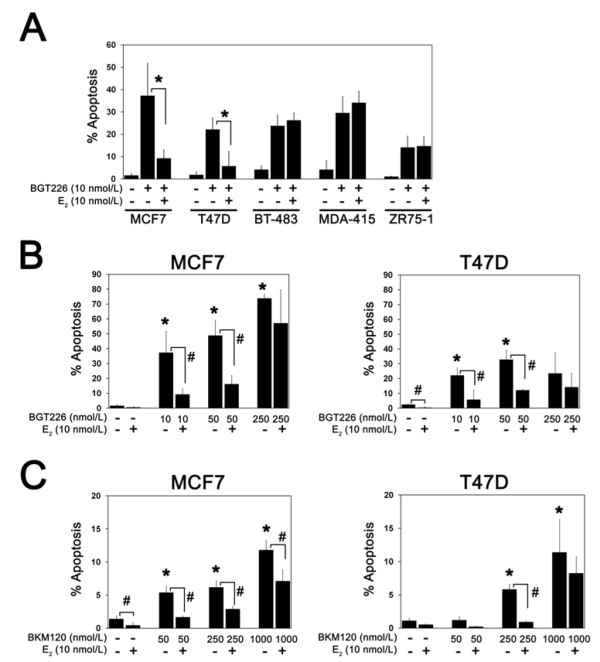
**Estrogen suppresses induction of apoptosis by phosphatidylinositol-3-kinase pathway inhibitors**. **(a) **Estradiol suppresses induction of apoptosis by BGT226. Cells cultured without or with 10 nmol/l 17β-estradiol (E_2_) were treated with 10 nmol/l BGT226, and apoptosis was measured after 4 days of treatment. Results are from at least three replicates for each treatment condition per cell line. Significant suppression of apoptosis by E_2 _is indicated (*P < 0.05). Suppression of phosphatidylinositol-3-kinase inhibitor-induced apoptosis by E_2_is dose dependent. Cells cultured without or with 10 nmol/l E_2 _were treated with the indicated concentrations of **(b) **BGT226 or **(c) **BKM120 and apoptosis was measured after 4 days of treatment. Results are from at least three replicates for each treatment condition per cell line. Significant induction of apoptosis in estrogen-deprived cells is indicated (*P < 0.05). Suppression of apoptosis by E_2 _in drug-treated and nontreated cells is indicated (^#^*P *< 0.05).

We have previously shown that estradiol significantly suppressed the induction of apoptosis by inhibition of p110α and p110β by RNA interference or treatment with the dual PI3K/mTOR inhibitor BEZ235 in ER-positive MCF7, T47D and HCC712 cells [5]. To determine whether estradiol broadly inhibits apoptosis induced by other PI3K inhibitors and in other ER-positive cell lines, the effect of BGT226 was compared in the presence and absence of estradiol. While estradiol suppressed BGT226-induced apoptosis in STED MCF7 and T47D cells, estradiol had no effect on PI3K inhibitor-induced apoptosis in BT-483, MDA-MB-415 and ZR75-1 cells (Figure 4a). Treatment with estradiol induced proliferation in these lines, however, suggesting that the ER was functional ([5] and data not shown). Dose escalation of BGT226 (Figure 4b) and BKM120 (Figure 4c) in MCF7 and T47D cells demonstrated that inhibition of cell death by estradiol was progressively lost at higher PI3K inhibitor concentrations. The modest increase in apoptosis with RAD001 treatment in STED MCF7 cells (Figure 3c) was also suppressed by estradiol (data not shown).

Overall, these data suggest estradiol-induced resistance is a shared characteristic across all three classes of PI3K pathway inhibitors tested, but there is marked heterogeneity in the inhibitory effect of estradiol across ER-positive breast cancer cell lines.

### BGT226, BKM120 and RAD001 inhibit PI3K pathway signaling despite long-term estrogen deprivation

**Figure 5:**
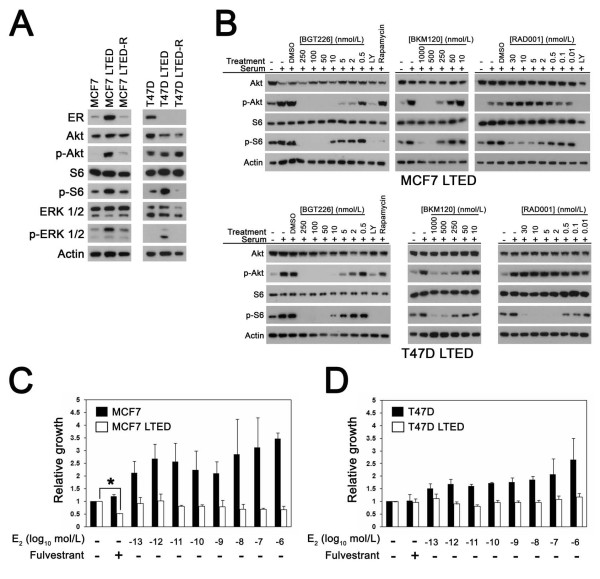
**Inhibition of phosphatidylinositol-3-kinase pathway signaling in estrogen-independent long-term estrogen-deprived cells**. **(a) **Western blot showing estrogen receptor (ER) expression and levels of p-Akt, p-S6 and p-ERK1/2 in estrogen-deprived parental MCF7 and T47D cells and in estrogen-deprived long-term estrogen deprivation (LTED) and estrogen-retreated long-term estrogen-deprived (LTED-R) sublines of each parental cell line. Equal amounts of total protein (25 μg) were immunoblotted for the indicated proteins. Total Akt, S6, ERK1/2 and actin are shown as loading controls. Representative results obtained from at least two different protein isolates per cell line are shown. **(b) **Western blot showing effects of BGT226, BKM120 and RAD001 on p-Akt and p-S6 in MCF7 LTED and T47D LTED lines. Representative results obtained in at least two experiments per cell line per drug treatment are shown. Estradiol (E_2_) stimulates the growth of MCF7 and T47D cells, but not **(c) **MCF7 LTED cells and **(d) **T47D LTED cells. Cells were treated with or without the indicated concentrations of E_2 _for 7 days followed by growth measurement. Cells were treated with fulvestrant (300 nmol/l) to directly inhibit ER function. Values are normalized to untreated cells. Shown are results from representative experiments performed in triplicate. Fulvestrant inhibited the growth of MCF7 LTED cells (*P < 0.05). DMSO, dimethylsulfoxide; LY, LY294002.

To model the effects of PI3K pathway inhibition in aromatase-inhibitor-resistant breast cancer cells, variants of the MCF7 and T47D lines were generated through LTED by over 9 months of culture in low-estrogen conditions (Figure 5a). ER upregulation and increased phosphorylation of Akt, S6 and the MAPK/ERKs (p-ERK) was observed in MCF7 LTED cells compared with the parental line. In the T47D LTED line, S6 and ERK phosphorylation, but not p-Akt, was higher than in parental T47D cells, and ER expression was downregulated to undetectable levels.

Both LTED lines were subsequently retreated with estradiol (10 nmol/l) for at least 4 months to determine whether estradiol re-exposure could reverse the signaling effects associated with LTED. In the resulting MCF7 revertant subline (MCF7 LTED-R), ER expression and levels of p-Akt, p-S6 and p-ERKs were downregulated to similar levels observed in the parental MCF7 cells, indicating that prolonged estradiol re-exposure reversed the effects of LTED on these proteins. In contrast, while S6 and ERK phosphorylation were downregulated by estradiol in T47D LTED-R cells, ER expression levels were not restored - at least not to a level detectable by western blot. The effect of the three PI3K pathway inhibitors on signal transduction demonstrated that the dose-response relationships for all three agents were similar to those observed in the parental MCF7 and T47D cell lines (Figure 5b). The sensitivity of the LTED lines to estradiol and fulvestrant was also determined. As expected, proliferation of MCF7 LTED and T47D LTED cells was not enhanced by increasing concentrations of estradiol (Figure 5c,d). Indeed the MCF7 LTED model was paradoxically inhibited by estradiol because 10 nmol/l treatment for >10 days inhibited growth and induced cell death [18,19] (data not shown). Treatment of estrogen-deprived MCF7 LTED with the ER-selective inhibitor fulvestrant [20] inhibited the growth of cells, demonstrating that ER remains functionally important for the growth of these cells despite the absence of supplemental estradiol. In contrast, treatment with estradiol or fulvestrant did not have significant effects on the growth of ER-negative T47D LTED cells (Figure 5d).

### Long-term estrogen-deprived cells are resistant to the induction of apoptosis by low-dose PI3K pathway inhibitors

**Figure 6:**
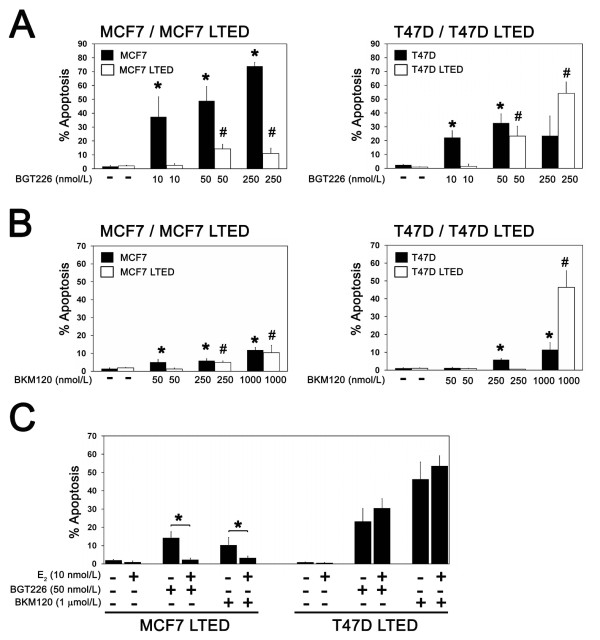
**Long-term estrogen-deprived cells are resistant to the induction of apoptosis by phosphatidylinositol-3-kinase pathway inhibitors**. Estrogen-deprived cells were treated with the indicated concentrations of **(a) **BGT226 or **(b) **BKM120 and apoptosis was measured after 4 days of treatment. Results are from at least three replicates for each treatment condition per cell line. Significant induction of apoptosis with PI3K inhibitors in parental (*P < 0.05) and long-term estrogen deprivation (LTED) lines (^#^*P *< 0.05) compared with untreated control is indicated. **(c) **Estrogen suppresses induction of apoptosis in LTED cells by phosphatidylinositol-3-kinase inhibitors. Cells cultured without or with estradiol were treated with BGT226 or BKM120 as indicated and apoptosis was measured after 4 days. Results are from at least three replicates for each treatment condition. Significant suppression of apoptosis by estradiol is indicated (*P < 0.05).

To determine the effect of LTED on PI3K drug sensitivity, we compared the ability of BGT226 and BKM120 to induce apoptosis in STED and LTED cell line pairs. In comparison with MCF7 and T47D STED cells, higher drug concentrations were required for both BGT226 (Figure 6a) and BKM120 (Figure 6b) to induce significant apoptosis under LTED conditions. The LC_50 _values for BGT226 in both LTED lines, and for BKM120 in T47D LTED cells, were consistent with resistance to apoptosis measured by TUNEL (Table 1). At the highest doses of BKM120 and BGT226 tested, however, T47D LTED cells were more sensitive than STED T47D cells; this pattern was not replicated in MCF7 LTED cells, where resistance to BGT226 persisted at all of the doses tested.

Despite resistance to the proliferative effects of estradiol, acute treatment with estradiol suppressed apoptosis induced by BGT226 and BKM120 treatment in MCF7 LTED cells -indicating that the survival effects of estradiol were decoupled from mitogenic effects (Figure 6c). In contrast, estradiol did not suppress BGT226-induced or BKM120-induced apoptosis in ER-negative T47D LTED cells.

### Treatment with fulvestrant sensitizes MCF7 LTED cells to PI3K inhibition

**Figure 7:**
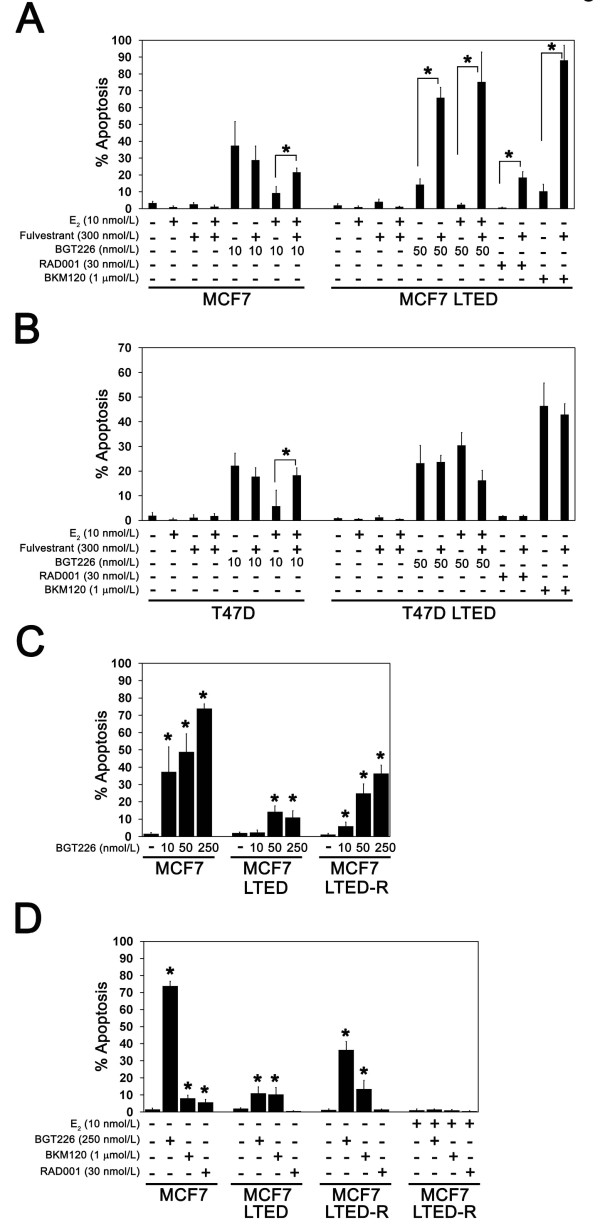
**Fulvestrant and estradiol treatment sensitize long-term estrogen-deprived cells to phosphatidylinositol-3-kinase inhibitors**. Estrogen-deprived **(a) **MCF7 and MCF7 long-term estrogen deprivation (LTED) cells or **(b) **T47D and T47D LTED cells were treated with fulvestrant, estradiol (E_2_) or phosphatidylinositol-3-kinase (PI3K) pathway inhibitors as indicated and apoptosis was measured after 4 days of treatment. Results are from at least three replicates for each treatment condition. Abrogation of E_2 _rescue or potentiation of PI3K inhibitor-induced apoptosis by fulvestrant is indicated (*P < 0.05). **(c) **Estrogen-deprived MCF7, MCF7 LTED and MCF7 estrogen-retreated long-term estrogen-deprived (LTED-R) cells were treated with the indicated concentrations of BGT226 and apoptosis was measured after 4 days of treatment. Results are from at least three replicates for each treatment condition. Significant activation of apoptosis is indicated (*P < 0.05). **(d) **MCF7, MCF7 LTED and MCF7 LTED-R cells were treated with E_2_, BGT226, BKM120 or RAD001 as indicated and apoptosis was measured after 4 days. Results are from at least three replicates for each treatment condition. Significant activation of apoptosis with respect to untreated control cells is indicated (*P < 0.05).

To model options for patients with disease progression on aromatase inhibitor treatment, the effect of fulvestrant was studied in LTED lines. Fulvestrant alone did not promote apoptosis in STED cells or LTED cells (Figure 7a,b); fulvestrant strongly potentiated apoptosis when combined with BGT226, BKM120 and RAD001 treatment in MCF7 LTED cells, however, confirming that ligand-independent ER activity promoted PI3K inhibitor resistance (Figure 7a). In contrast, treatment with fulvestrant did not promote apoptosis in the ER-negative T47D LTED cells with any of the three agents tested.

Taken together, these data suggest that fulvestrant may sensitize cells to the therapeutic effects of PI3K inhibitors under circumstances where resistance to estrogen deprivation is associated with ligand-independent ER activity.

### Prolonged retreatment with estradiol re-sensitizes MCF7 LTED cells to PI3K inhibition

As an alternative to fulvestrant, breast cancer patients with advanced ER-positive aromatase-inhibitor-resistant disease can be treated with low-dose estradiol to induce tumor regression and, in some instances, resensitize the patients' tumor to estrogen deprivation therapy with an aromatase inhibitor [21]. The MCF7 LTED line provides an *in vitro *parallel of these clinical findings because, when these cells are re-exposed to estradiol, cell growth slows dramatically, followed by a period of recovery during which cell growth once again becomes estrogen dependent (MCF7 LTED-R) (data not shown).

To determine whether MCF7 LTED-R cells also recovered sensitivity to PI3K inhibition, the effects of BGT226, BKM120 and RAD001 treatment were compared between MCF7 LTED-R cells and MCF7 LTED cells (Figure 7c,d). Consistent with partial recovery of sensitivity to PI3K inhibition, lower doses of BGT226 were able to induce apoptosis in estrogen-deprived MCF7 LTED-R cells in comparison with MCF7 LTED cells (Figure 7c). In contrast, the levels of cell death with BKM120 (1 μmol/l) were similar in all three MCF7 cell line variants (Figure 7d) and sensitivity to RAD001 was lost in MCF7 LTED-R cells despite reintroduction of estrogen deprivation.

### *PIK3CA *mutations are common in relapsed ER-positive breast cancer

The *in vitro *studies described above suggested that a combination of fulvestrant and a PI3K pathway inhibitor may be an effective approach for aromatase-inhibitor-resistant advanced breast cancer, particularly in *PI3KCA *mutant cases that are persistently ER-positive at relapse. Since *PIK3CA *mutation has been reported to be associated with a more favorable prognosis [7], however, it was unclear how many patients with ER-positive *PIK3CA *mutant breast cancer would present with advanced disease. Fresh-frozen research biopsies were therefore obtained from 51 patients with recurrent or metastatic disease for *PIK3CA *mutation testing (Table 2). Their median age at initial cancer diagnosis was 53.4 (32.3 to 79.9) years. The median follow-up was 51.7 (0.9 to 256.7) months. Forty-three out of the 51 (84.3%) patients were deceased at the time of analysis. At initial diagnosis, 32 tumors were ER-positive, 17 tumors were ER-negative, and two tumors were of unknown status. Five out of the 32 ER-positive tumors changed to ER-negative status at recurrence.

**Table 2:** Clinical characteristics of the 51 recurrent or metastatic breast cancers

Characteristic at initial diagnosis	Number of patients	%
Total	51	100
Median age (years)	53.4 (32 to 79)	
Median follow-up time (months)	51.7 (0.9 to 256)	
Stage		
I to III	34	67
IV	17	33
Estrogen receptor		
Positive	32	63
Negative	17	33
Missing	2	4
Human epidermal growth factor receptor 2		
Positive	15	29
Negative	33	65
Missing	3	6
*PIK3CA *mutation	16	31

**Figure 8:**
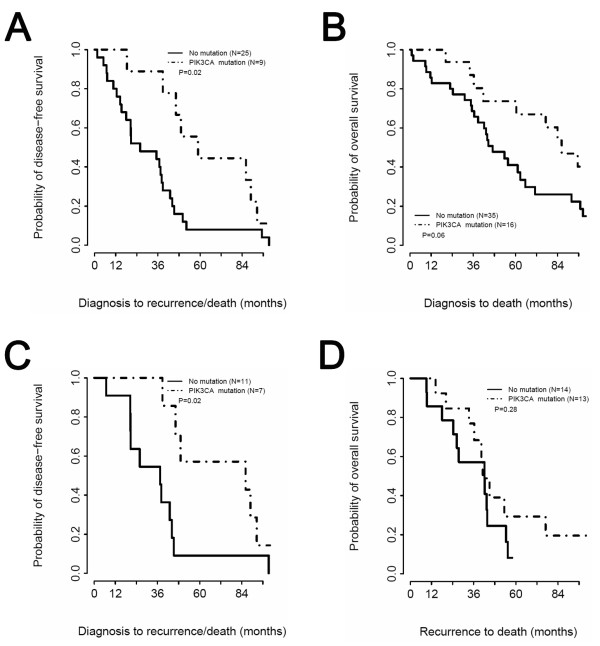
***PIK3CA *mutation associates with superior disease-free survival**. **(a) **Kaplan-Meier curves for disease-free survival (DFS) based on *PIK3CA *mutation status in all patients. **(b) **Kaplan-Meier curves comparing overall survival (OS) in all patients with *PIK3CA *wild-type and mutant tumors. **(c) **Kaplan-Meier curves for DFS based on *PIK3CA *mutation status in patients with estrogen receptor (ER)-positive breast cancer. **(d) **Kaplan-Meier curves comparing OS in ER-positive breast cancer patients (ER status at time of recurrence) with *PIK3CA *wild-type and mutant tumors, from the time of relapse.

*PIK3CA *mutation analysis was performed on the 27 ER-positive and 24 ER-negative recurrent specimens. We included both ER-positive and ER-negative cases to interrogate the relationship between *PIK3CA *mutation and ER status in the recurrent disease population. A *PIK3CA *mutation was identified in 16 of the 51 tumors (31.4%; eight in the helical domain, eight in the kinase domain), a prevalence similar to that observed in studies that examined primary breast cancer tissue [6,7,22]. *PIK3CA *mutation was strongly associated with ER positivity (*P *= 0.0076). Among the 27 ER-positive tumors, 13 (48%) were *PIK3CA *mutant. In contrast, only three of the 24 ER-negative tumors were *PIK3CA *mutant. ER expression was maintained in 13 out of 14 cases with *PIK3CA *mutation (Table 3). Consistent with previous reports [7], *PIK3CA *mutation was associated with a later relapse pattern (disease-free survival *P *= 0.02, Figure 8a), with a trend for patients with *PIK3CA *mutant disease exhibiting a lower mortality rate (overall survival *P *= 0.06, Figure 8b). In an analysis restricted to patients with initially ER-positive disease, *PIK3CA *mutant cases still relapsed later than nonmutant cases (disease-free survival *P *= 0.02, Figure 8c). Survival after relapse in persistently ER-positive tumors (a potentially important endpoint for drug approval), however, was not different between *PIK3CA *wild-type and mutant cases, although the very small sample size meant that only very large effects could have been detected (Figure 8d).

**Table 3:** *PIK3CA *correlation analysis

	*PIK3CA *mutant	*PIK3CA *wild-type	*n*	*P *value
ER at diagnosis				
Positive	14	18	32	0.0082
Negative	1	16	17	
HER2 at diagnosis				
Positive	2	13	15	0.17
Negative	12	21	33	
ER at recurrence				
Positive	13	14	27	0.0076
Negative	3	21	24	
HER2 at recurrence				
Positive	1	11	12	0.79
Negative	14	24	38	

ER, estrogen receptor; HER2, human epidermal growth factor receptor 2.

### Discussion

The primary aim of the present study was to assess the case for combined targeting of ER and PI3K pathway inhibition by examining an extended panel of ER-positive breast cancer cell lines using clinical grade PI3K and ER pathway inhibitors. Conclusions focused on the induction of apoptosis because the ability of PI3K inhibitors to induce cell death, rather than inhibit cell proliferation, is considered to be the best predictor of *in vivo *anti-tumor response [17]. The dual PI3K/mTOR inhibitor BGT226 generally produced the highest levels of apoptosis when combined with estrogen deprivation in sensitive cells, followed by the PI3K isoform selective inhibitor BKM120. In contrast, the level of apoptosis induced by the mTOR-selective inhibitor RAD001 in estrogen-deprived cells was modest by comparison, even in the most sensitive cells. Poor induction of apoptosis by RAD001 in estrogen-deprived ER-positive cells is consistent with the results of a randomized phase 2 trial (NCT00107016) that evaluated the efficacy of the aromatase inhibitor letrozole and RAD001 as neoadjuvant treatment for ER-positive breast cancer. Despite greater inhibition of tumor proliferation, the pathological complete response rate was not increased by RAD001 over that observed using letrozole alone - suggesting no clinically significant increase in cell death was achieved [23]. Our data suggest that if tolerable at active doses, direct inhibitors of PI3K might be more effective in this setting.

The sensitizing effect of *PIK3CA *mutation to the dual PI3K/mTOR inhibitor BEZ235 and to a selective Akt inhibitor in breast cancer cells has already been reported [9,17]. These studies included few *PIK3CA *wild-type ER-positive HER2-negative cells, however, and it was not clear how *PIK3CA *mutation impacts PI3K inhibitor sensitivity in the setting of estrogen deprivation. Our data support the conclusion that *PIK3CA *mutation confers sensitivity to PI3K pathway inhibitors in the setting of new agents in clinical development and that this differential effect is maintained under estrogen-deprived conditions. However, the impact of estradiol on PI3K pathway inhibitor activity in *PIK3CA *mutant cells was not uniform. Estradiol suppressed apoptosis induced by BGT226 in MCF7 and T47D cells but not in BT-483 cells. The identification of additional biomarkers will probably therefore be necessary to fully predict the efficacy of PI3K/endocrine combination therapy in *PIK3CA *mutant ER-positive tumors. Consistent with previous reports, the effect of *PTEN *mutation on the sensitivity of ER-positive cells to PI3K inhibitors also appears complex [9,17]. Whereas the PTEN-negative MDA-MB-415 and ZR75-1 lines were sensitive to both BGT226 and BKM120, the CAMA-1 line, which is PTEN mutant but does express low amounts of PTEN, was resistant to both inhibitors. The reasons for the inconsistent effects of PTEN deficiency on PI3K pathway inhibitor sensitivity in ER-positive cells will also require further study.

Estradiol is thought to prevent apoptosis through plasma-membrane-initiated or nongenomic signaling by the ER through activation of the PI3K and MAPK pathways [24,25]. Consistent with these reports, our results indicate that transduction of the estradiol survival signal increases PI3K inhibitor dose requirements in some ER-positive breast cancer cells (for example, MCF7 and T47D cells) but not others (BT-483, MDA-MB-415 and ZR75-1 cells). Interestingly, our results also show that the anti-apoptotic activity of estradiol is preserved in breast cancer cells that do not require estradiol for proliferation as a consequence of prolonged estrogen deprivation (Figure 6). The decoupling of the proliferative and anti-apoptotic effects of estrogen suggests that continuing estrogen deprivation in progressing patients and adding a PI3K inhibitor might be a strategy worth testing.

The optimal endocrine combination with PI3K inhibition in cells resistant to estrogen deprivation is a critical consideration since the overwhelming majority of patients with advanced breast cancer have already been treated with an aromatase inhibitor in the adjuvant setting. Treatment options include an anti-estrogen (such as the ER downregulator fulvestrant) [26] or therapy with low-dose estradiol [21]. We modeled these second-line approaches in contrasting LTED cell lines, one where ER expression was maintained and one where it was lost, in order to reflect the clinical observation that upon disease progression ER is downregulated in a proportion of cases [27,28]. Both LTED lines were found to be relatively resistant to PI3K inhibitors compared with the parental lines, consistent with reports that acquiring the ability to grow in the absence of estrogen is associated with increased PI3K and MAPK signaling [29]. The use of fulvestrant efficiently sensitized MCF7 LTED cells to both BKM120 and BGT226, however, consistent with a key role for ligand-independent ER activity in PI3K inhibitor resistance. The use of estradiol to revert the LTED phenotype, followed by re-institution of estrogen deprivation, is a viable alternative strategy; however, the restoration of sensitivity to PI3K inhibition with this approach appeared less profound than with fulvestrant treatment.

Taken together our data provide a rationale for combining estrogen deprivation with PI3K inhibitors for the treatment of *PIK3CA *mutant estrogen-dependent, ER-positive tumors and for the combination of fulvestrant with PI3K inhibitors in patients with ER-positive, aromatase-inhibitor-resistant disease. However, further studies will be necessary to effectively translate these preclinical data into the clinical setting. These studies could include additional preclinical modeling in *PIK3CA *wild-type estrogen-deprivation-resistant tumor lines to determine whether *PIK3CA *mutation is necessary in endocrine-resistant tumors to confer PI3K inhibitor sensitivity. In addition, incorporating biomarker (*PIK3CA *mutation, Ki67 tumor cell proliferation and cell death markers) analysis in early-phase PI3K inhibitor trials may aid in identifying patients most likely to benefit from these therapeutic agents.

To address the prevalence of the target population for a fulvestrant/PI3K inhibitor trial for second-line treatment of ER-positive *PIK3CA *mutant relapsed disease, we analyzed 51 advanced disease biopsies from both ER-positive and ER-negative cases for *PIK3CA *mutation and correlated findings with the clinical trajectory of the patients. While patients with ER-positive *PIK3CA *mutant tumors tended to relapse later than patients with ER-negative or ER-positive *PIK3CA *wild-type tumors, the *PIK3CA *mutation prevalence in ER-positive relapsed disease was high (approximately 50%). These findings are consistent with those recently reported by Dupont Jensen and colleagues on an analysis of 104 paired primary and metastatic breast tumors [30]. In this study, *PIK3CA *mutation was detected in 53% of the metastatic tumors and 45% of the primary tumors, indicating an apparent net gain in *PIK3CA *mutation in metastatic disease that was thought to be due to heterogeneity in the primary tumor. The high prevalence of *PIK3CA *mutation in metastatic or recurrent breast cancer suggests that PI3K-pathway-targeted therapeutics will be clinically relevant in this setting. These data also indicate that analysis of the recurrent disease will be necessary for selection of patients based upon tumor *PIK3CA *mutation status.

## Conclusions

Estrogen-dependent, ER-positive breast cancers with *PIK3CA *mutation and, possibly, PTEN loss will be most responsive to PI3K isoform inhibitors in combination with estrogen deprivation therapy. By increasing tumor cell death, these combinations may be sufficient to eradicate ER-positive cells - thereby preventing acquired endocrine resistance. When estrogen derivation resistance and relapse does occur in *PIK3CA *mutant ER-positive cells, fulvestrant combined with PI3K inhibition may be an effective salvage approach - and screening of relapse biopsies for *PIK3CA *mutation confirms that a population of patients who meet these criteria is easy to identify.

## Abbreviations

CSS: charcoal-stripped serum; ER: estrogen receptor; ERK: extracellular signal-regulated kinase; FBS: fetal bovine serum; HER2: human epidermal growth factor receptor 2; IC_50_: half maximal inhibitory concentration; LC_50_: 50% lethal concentration; LTED: long-term estrogen deprivation; LTED-R: estrogen-retreated long-term estrogen-deprived; MAPK: mitogen-activated protein kinase; mTOR: mammalian target of rapamycin; p-Akt: phospho-Akt; p-ERK: phospho-ERK; PI3K: phosphatidylinositol-3-kinase; *PI3KCA*: phosphoinositide-3-kinase, catalytic, α-polypeptide; p-S6: phospho-Ser235/236 S6; PTEN: phosphatase and tensin homolog; siRNA: small interfering RNA; STED: short-term estrogen deprivation; TUNEL: Terminal deoxynucleotidyl transferase-mediated nick-end labeling.

## Competing interests

MJE has received honoraria and grants and has served as a consultant for AstraZeneca, Novartis and Pfizer. No pharmaceutical company funding was received for this project. The authors declare that they have no competing interests.

## Authors' contributions

CGS, CXM, RJC and MJE contributed to the experimental design. CGS, CXM, RJC, CP and LL were responsible for performing experiments and data analysis. TG coordinated sample collection of patients with recurrent and metastatic breast cancer. FG was responsible for statistical analysis of tumor samples from advanced breast cancer patients. CGS, CXM, RJC, and MJE contributed to manuscript preparation.
